# Acute alcohol consumption increases systemic endotoxin bioactivity for days in healthy volunteers—with reduced intestinal barrier loss in female

**DOI:** 10.1007/s00068-021-01666-4

**Published:** 2021-04-11

**Authors:** Ramona Sturm, Florian Haag, Andrea Janicova, Baolin Xu, Jan Tilmann Vollrath, Katrin Bundkirchen, Ildiko Rita Dunay, Claudia Neunaber, Ingo Marzi, Borna Relja

**Affiliations:** 1 Department of Trauma, Hand and Reconstructive Surgery, Goethe University, Frankfurt, Germany; 2 Experimental Radiology, Department of Radiology and Nuclear Medicine, Otto Von Guericke University, Magdeburg, Germany; 3 Trauma Department, Hannover Medical School, Hannover, Germany; 4 Institute of Inflammation and Neurodegeneration, Otto Von Guericke University, Magdeburg, Germany

**Keywords:** Alcohol, Barrier, FABP, SCD14, Syndecan-1, Gender

## Abstract

**Objective:**

Trauma is the most common cause of death among young adults. Alcohol intoxication plays a significant role as a cause of accidents and as a potent immunomodulator of the post-traumatic response to tissue injury. Polytraumatized patients are frequently at risk to developing infectious complications, which may be aggravated by alcohol-induced immunosuppression. Systemic levels of integral proteins of the gastrointestinal tract such as syndecan-1 or intestinal fatty acid binding proteins (FABP-I) reflect the intestinal barrier function. The exact impact of acute alcohol intoxication on the barrier function and endotoxin bioactivity have not been clarified yet.

**Methods:**

22 healthy volunteers received a precisely defined amount of alcohol (whiskey–cola) every 20 min over a period of 4 h to reach the calculated blood alcohol concentration (BAC) of 1‰. Blood samples were taken before alcohol drinking as a control, and after 2, 4, 6, 24 and 48 h after beginning with alcohol consumption. In addition, urine samples were collected. Intestinal permeability was determined by serum and urine values of FABP-I, syndecan-1, and soluble (s)CD14 as a marker for the endotoxin translocation via the intestinal barrier by ELISA. BAC was determined.

**Results:**

Systemic FABP-I was significantly reduced 2 h after the onset of alcohol drinking, and remained decreased after 4 h. However, at 6 h, FABP-I significantly elevated compared to previous measurements as well as to controls (*p* < 0.05). Systemic sCD14 was significantly elevated after 6, 24 and 48 h after the onset of alcohol consumption (*p* < 0.05). Systemic FABP-I at 2 h after drinking significantly correlated with the sCD14 concentration after 24 h indicating an enhanced systemic LPS bioactivity. Women showed significantly lower levels of syndecan-1 in serum and urine and urine for all time points until 6 h and lower FABP-I in the serum after 2 h.

**Conclusions:**

Even relative low amounts of alcohol affect the immune system of healthy volunteers, although these changes appear minor in women. A potential damage to the intestinal barrier and presumed enhanced systemic endotoxin bioactivity after acute alcohol consumption is proposed, which represents a continuous immunological challenge for the organism and should be considered for the following days after drinking.

## Introduction

Trauma is the leading cause of death among young adults [1, 2], while alcohol use accounts as a major cause of accidents [3]. Among alcohol-related accidents, road traffic injuries are leading, followed by self-harm, interpersonal violence and falls [4]. In Germany, a blood alcohol level of 0.5 per mille is currently permitted in road traffic to prevent drink-driving [5]. The limit value of 0.5 and 0.8 per mille is frequently used in international comparisons, whereas some countries have no limitation of blood alcohol concentration in road traffic [6]. The percentage of alcoholised polytraumatized patients, depending on the study, averages at > 25% [7]. Patients who initially survive an acute and relevant blood loss, massive tissue injury, or severe traumatic brain injury (TBI) are at high risk for developing post-traumatic imbalance of immunological mediators and immune cells, which may result in inflammatory complications, such as sepsis or multiple organ failure [8, 9]. In alcoholised polytraumatized patients and alcoholised patients with TBI reduced leukocyte numbers and lowered systemic interleukin (IL)-6 levels [7, 10] indicating an immunosuppressive effect of alcohol have been reported. In this context, damaged intestinal barrier and alcohol use play also decisive roles [11]. The mortality after surgical interventions is increased in severely traumatized patients with acute alcohol intoxication [12]. Studies indicate that acute alcohol intoxication reduces the lipopolysaccharide (LPS)-induced production and release of proinflammatory cytokines and contributes to the development of Toll-like receptor (TLR)4/endotoxin (LPS) tolerances in the murine approach [13].

As a result of damaged enterocytes and thus the intestinal barrier after, e.g., trauma or hypoperfusion (shock), membrane and intracellular proteins are released into the extracellular space and subsequently into the circulation and urine [14]. Furthermore, a damaged intestinal barrier promotes the translocation of damage-associated molecular pattern or bacteria as well. Fatty acid binding proteins (FABPs) are a group of nine partially tissue-specific transport proteins that are localized intracellularly or in the cytoplasmic membrane of different cells [15]. The FABP-I or FABP2 are specifically expressed in enterocytes in the small intestine and occasionally in the colon [16]. Recently, it was shown that FABP-I can be used as an early biomarker for the detection of abdominal injuries in general and specifically for intestinal injuries [17]. Elevated FABP-I level evaluated early after trauma depends on the tissue injury pattern and the presence of shock as well, which is associated with a barrier breakdown [18]. Moreover, the FABP-I in urine and serum may serve as a biomarker for diagnosis of acute mesenteric ischemia [19, 20] and can predict mortality and bowel ischemia in patients with septic shock [21]. Syndecan-1 (CD138) as a transmembrane heparan sulphate proteoglycan is expressed predominantly on the basolateral surface of epithelial cells and plasma cells [22]. As a transmembrane protein with extracellular and cytoplasmic domains, syndecane-1 is involved in cell–matrix interactions with cell binding, cell migration and cytoskeletal organization as well as in cell proliferation and cell signalling [23]. In severely injured patients, syndecan-1 has been shown to be elevated in serum associated with subsequent sepsis [24]. CD14 is a surface antigen on monocytes and macrophages and exists in a membrane-bound or soluble form. The soluble (s)CD14 binds LPS and transfers it to the membrane-bound CD14, which serves as a co-receptor for the TLR4-receptor complex. Moreover, sCD14 can transfer the LPS directly to the TLR4-receptor complex [25, 26]. Furthermore, sCD14 seems to neutralize LPS by binding and limiting the amount of monocyte-bound LPS. This may lead to a reduction of the inflammatory response [27]. Soluble CD14 can be used as a biomarker for the LPS bioactivity. Following traumatic injury, intestinal bacteria or bacterial components (e.g., LPS) can translocate into blood circulation where they trigger a proinflammatory response [28].

Chronic alcohol consumption alters the intestinal flora and can further lead to a malfunction and intestinal hyperpermeability [29]. Little is known about the direct impact of acute alcohol consumption on the intestinal barrier, permeability and effects of the immune system, in particular the differences between men and women are unknown.

Since acute alcohol consumption induces immunosuppression and is associated with infectious complications, the aim of the present study was to evaluate the dose- and time-dependent effects of acute alcohol consumption on the intestinal barrier and the immunological changes.

## Patients and methods

### Ethics

This study was performed in accordance with the institutional ethics committee approval (255/14) from the University Hospital of the Goethe University Frankfurt, in accordance with the Declaration of Helsinki and following the Strengthening the Reporting of Observational studies in Epidemiology-guidelines [30]. All healthy volunteers signed the written informed consent form accordance to the ethical standards after detailed explanation of the procedure, effects and objectives of the investigations.

### Study population

Twelve female und ten male healthy volunteers between 18 and 50 years were enrolled. Exclusion criteria were chronic alcohol consumption, pre-existing immunological disorders, chronic inflammatory and explicitly chronic intestinal diseases, HIV and infectious hepatitis or immune-suppressive medication. A detailed alcohol anamnesis was taken, including the standardized “Alcohol Use Disorders Identification Test” to exclude regular and chronic alcohol consumption. Furthermore, hepatic and renal insufficiencies were previously excluded by blood examination.

**Fig. 1:**
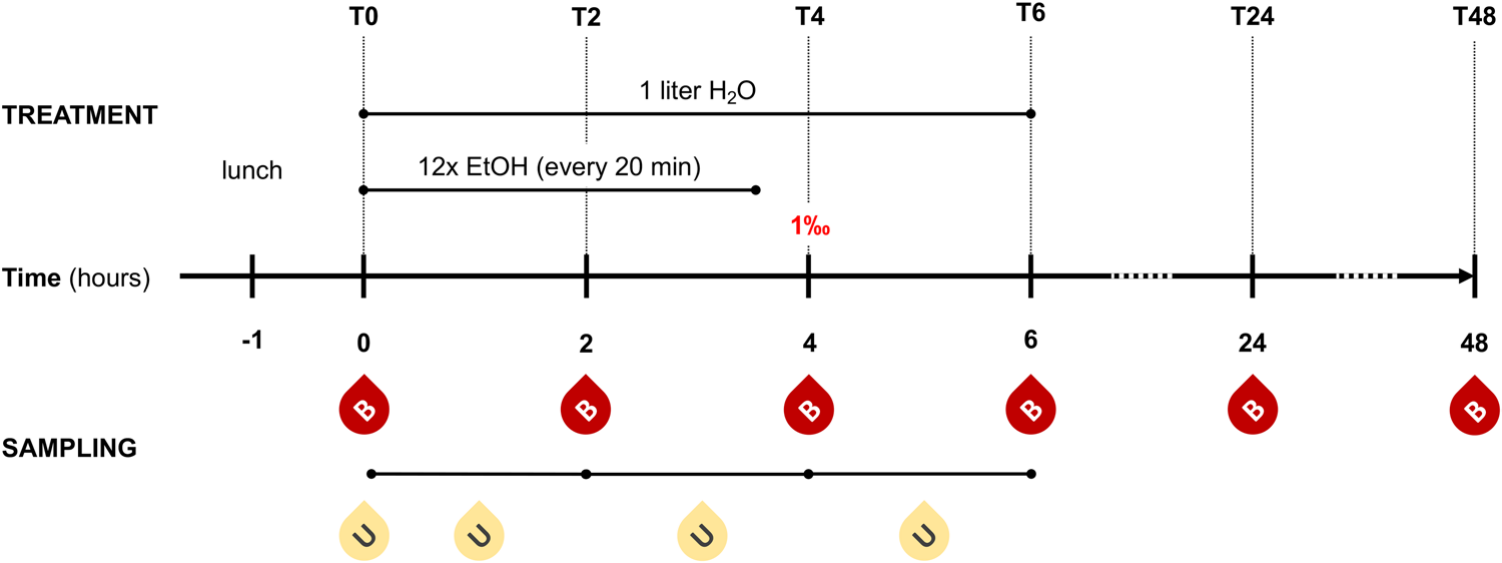
Experimental design. Before alcohol (ethanol, EtOH) drinking (T0), 2 h (T2), 4 h (T4), 6 h (T6), 24 h (T24) and 48 h (T48) after start of alcohol consumption sampling of either blood (B) or urine (U) from healthy volunteers was performed. The aim blood alcohol concentration of one per mille (1‰) was reached at T4

Healthy volunteers, who received a standardized lunch 1 hour before the experiment, drank an individually calculated amount of alcohol, which should lead to a blood alcohol level of 1‰ after the end of consumption. The calculation was made according to the modified Widmark equation depending on age, sex, height and weight. Every 20 min over 4 h equal mixed drinks consisting of whisky (Tennessee Whiskey Jack Daniels, 40%) and cola (Coca-Cola) in a mixing ratio of 1:2 were drunk. Subsequently, a 2-h monitoring phase without further alcohol consumption followed. All study participants drank 1 l of water during the first 6 h of the experiments. The experimental design is shown in Fig. 1.

### Blood sampling

Blood samples were taken before alcohol consumption (T0) as a control and after 2 h (T2), 4 h (T4), 6 h (T6), 24 h (T24) and 48 h (T48) after beginning of alcohol consumption. The blood was withdrawn in serum-gel tubes (Sarstedt, Nürmbrecht, Germany) and centrifuged immediately after collection at 2,000 × *g* for 15 min at 4 °C. The supernatant was stored at − 80 °C until analysis. Furthermore, the serum alcohol concentration was determined by the clinical laboratory.

### Urine sampling

Before starting the experiment, urine was delivered, centrifuged and frozen at − 80 °C (at T0). From T0 to T2, as well as from T2 to T4 and from T4 to T6, the urine was collected in separate containers, centrifuged at 2,000 × *g* for 15 min at 4 °C and stored at − 80 °C.

### Measurement of FABP-I, syndecan-1 and sCD14

Both serum and urine samples were thawed for direct use and measurement of FABP-I, syndecan-1 and sCD14. According to manufacturer's instructions, Human FABP2/I-FABP DuoSet enzyme-linked immunosorbent assay (ELISA) (R and D Systems, Minneapolis, USA, # DY3078) was used to measure intestinal FABP and Syndecan-1 DuoSet ELISA (R and D Systems, Minneapolis, USA, #DY2780) was used to measure syndecan-1 concentrations in serum and urine. Human CD14 DuoSet ELISA (R&D Systems, Minneapolis, USA, #DY383) was utilized according to manufacturer`s instructions to measure sCD14 concentrations in serum and urine.

### Statistical analysis

GraphPad Prism 6.0 software (GraphPad Software Inc. San Diego, CA, USA) was used to perform the statistical analysis. Data are given as mean ± standard error of the mean (SEM). The Kruskal–Wallis test with a Dunn’s post hoc test was applied to compare the differences between the groups. Spearman’s correlation coefficient was calculated to determine correlations. A *p* value below 0.05 was considered statistically significant.

## Results

### Study population

Twenty-two healthy volunteers were enrolled in this study. The mean age was 25 ± 4 years. 10 out of 22 patients were male (45.45%). The experimental design is shown in Fig. 1.

### Blood alcohol concentration

**Fig. 2:**
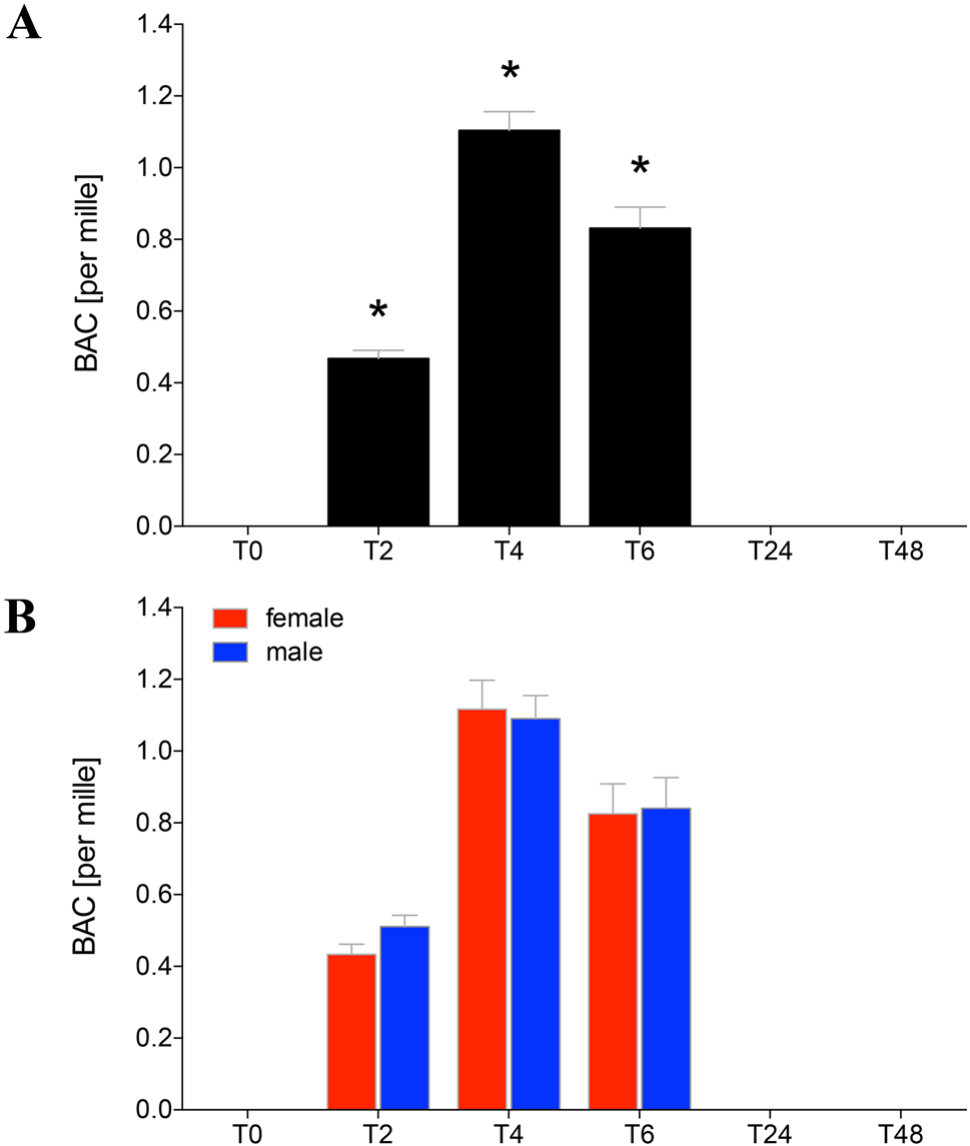
Blood alcohol concentration (BAC) in sera of healthy volunteers before, during and after alcohol drinking. Mean BAC as per mille determined in sera from healthy volunteers before (T0), 2 h (T2), 4 h (T4), 6 h (T6), 24 h (T24) and 48 h (T48) after start of alcohol consumption are given. **a** Data from all healthy volunteers (*n* = 22), and **b** gender-specific results are shown (female: *n* = 12 and male: *n* = 10). The data are presented as mean ± standard error of the mean. *p < 0.05 vs. T0

All volunteers had no measurable blood alcohol concentration at the beginning of the experiment. At T2 the legally permissible alcohol level (0.5‰) for the road traffic had nearly been reached. The BAC increased significantly at T2 to 0.46 ± 0.02‰ compared to T0 (*p* < 0.05, Fig. 2a). After T4, the aim concentration with 1.11 ± 0.05‰ was reached (Fig. 2a). 2 h after the end of alcohol consumption (T6), the BAC dropped to 0.83 ± 0.06‰ still being significantly enhanced compared to T0 (*p* < 0.05, Fig. 2a). At T24 and T48, no BAC was detectable. There were no significant gender-specific differences among the BAC levels (Fig. 2b).

### FABP-I in serum and urine

**Fig. 3:**
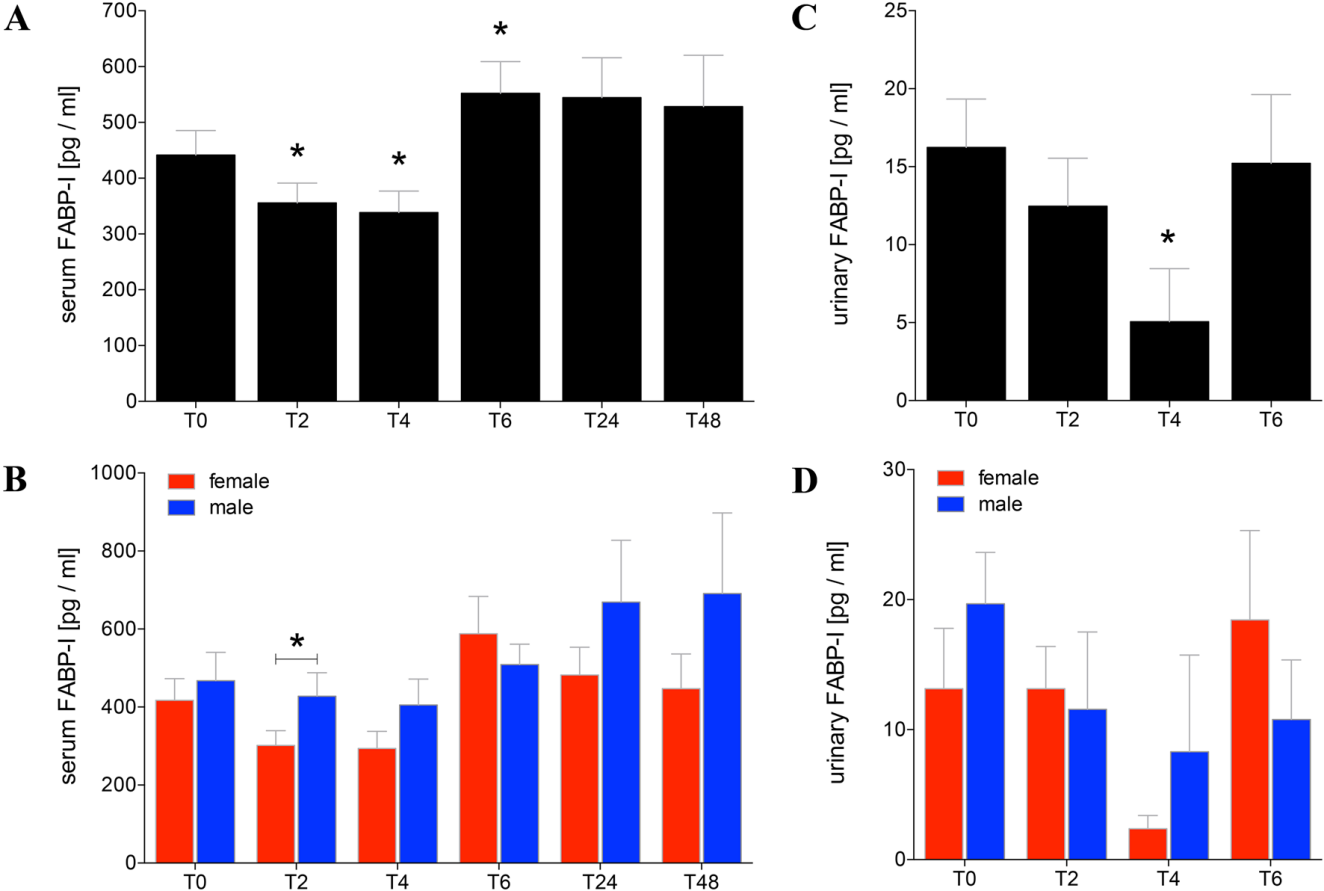
Levels of intestinal fatty-acid binding protein (FABP-I) in sera and urine of healthy volunteers before, during and after alcohol consumption. Mean values of intestinal FABP in pg/ml determined in sera (**a**, **b**) or urine (**c**, **d**) from healthy volunteers before (T0), 2 h (T2), 4 h (T4), and 6 h (T6) after start of alcohol consumption are given. 24 h (T24) and 48 h (T48) after experimentation, FABP-I was determined in sera as well. Urine samples were analysed before alcohol consumption (T0), collected and pooled over the first 2 h (T2), from T2 until the following 2 h (T4), and from T4 until T6 (T6). **a**, **c** Data from all healthy volunteers (*n* = 22) are shown, and **b**, **d** gender-specific results are shown (female: *n* = 12 and male: *n* = 10). The data are presented as mean ± standard error of the mean. *p < 0.05 vs. T0 or in B vs. indicated groups

Before alcohol intake, the intestinal FABP in serum of healthy volunteers was 441.30 ± 44.15 pg/ml (Fig. 3a). At T2 and T4 after beginning of acute alcohol consumption, the FABP-I in serum was significantly reduced compared to T0 (T2: 355.90 ± 35.44 or T4: 338.50 ± 38.40 vs. T0: 441.30 ± 44.15 pg/ml, *p* < 0.05, Fig. 3a). At T6, the intestinal FABP was significantly increased compared to T0 (T6: 552.20 ± 56.83 vs. T0: 441.30 ± 44.15 pg/ml, *p* < 0.05, Fig. 3a). The levels of intestinal FABP remained elevated for the next 2 days at T24 and T48, however, no statistical difference was found. Gender-specific analyses showed a significant difference between men and women at T2 with higher levels in men (*p* < 0.05, Fig. 3b).

The urinary level of 16.24 ± 3.90 pg/ml of intestinal FABP has been detected in healthy volunteers before alcohol consumption (Fig. 3c). In the collected urine at T4, the FABP-I concentration was significantly reduced compared to T0 (T4: 5.06 ± 3.40 vs. T0: 16.24 ± 3.90 pg/ml, *p* < 0.05, Fig. 3c). At T6, the FABP-I levels increased to normal values (Fig. 3c). The gender-specific analyses have shown no differences between men and women over the observational time course (Fig. 3d).

### Syndecan-1 in serum and urine

**Fig. 4:**
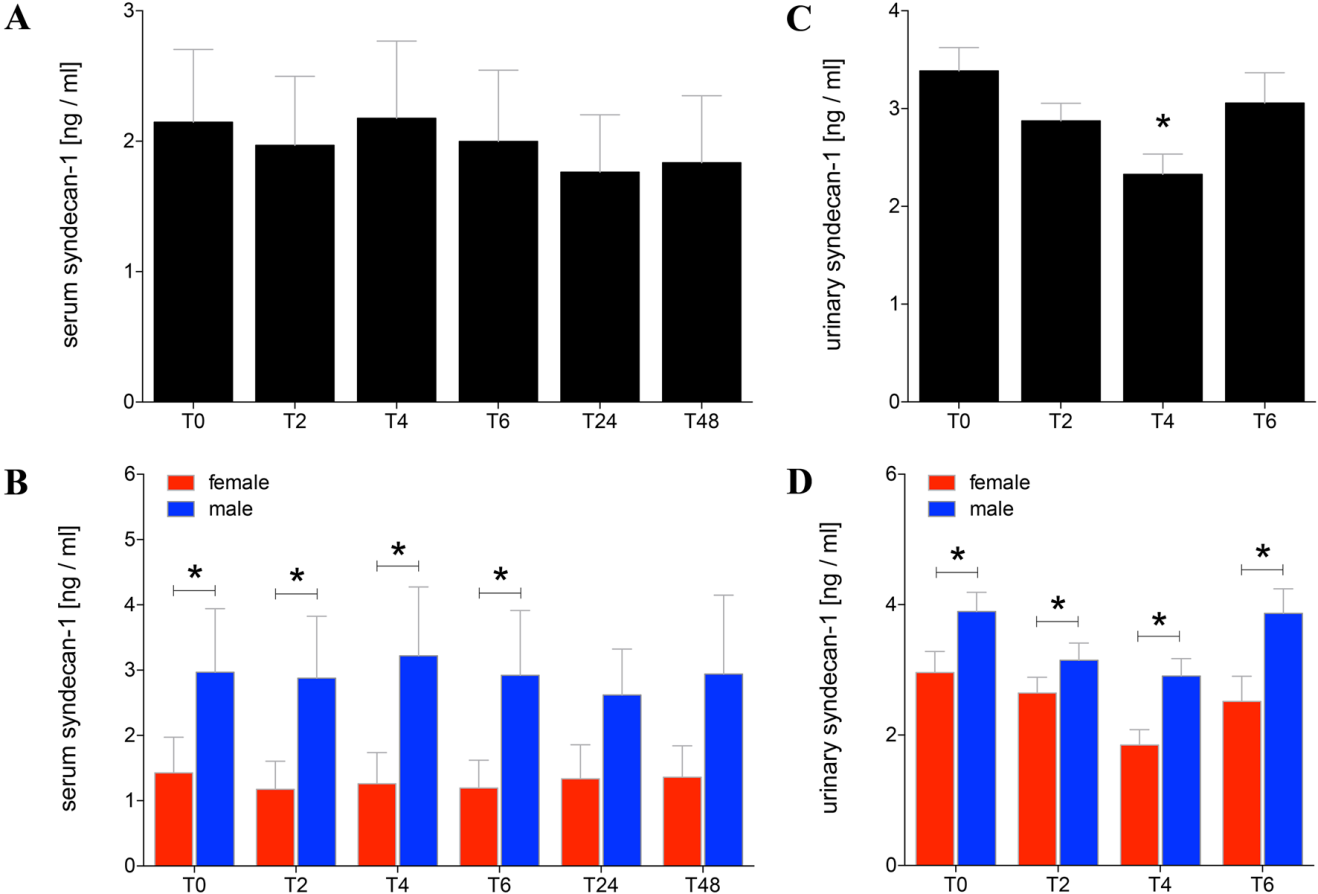
Syndecan-1 levels in sera and urine of healthy volunteers before, during and after alcohol consumption. Mean values of syndecan-1 in ng/ml determined in sera (**a**, **b**) or urine (**c**, **d**) from healthy volunteers before (T0), 2 h (T2), 4 h (T4), and 6 h (T6) after start of alcohol consumption are given. 24 h (T24) and 48 h (T48) after experimentation, syndecan-1 was determined in sera as well. Urine samples were analysed before alcohol consumption (T0), collected and pooled over the first 2 h (T2), from T2 until the following 2 h (T4), and from T4 until T6 (T6). **a**, **c** Data from all healthy volunteers (*n* = 22) are shown, and **b**, **d** gender-specific results are shown (female: *n* = 12 and male: *n* = 10). The data are presented as mean ± standard error of the mean. *p < 0.05 vs. T0 or in **b**, **d** vs. indicated groups

Beside in healthy volunteers, there were no significant differences among the serum concentrations of syndecan-1 during the observational period compared to normal values detected at T0 before alcohol administration (2.15 ± 0.56 ng/ml, Fig. 4a). However, the gender-specific values were significantly higher in men compared to women during the observational period between time points T0 and T6 (*p* < 0.05, Fig. 4b).

The urine levels of syndecan-1 before alcohol consumption at T0 were at 3.39 ± 0.24 ng/ml (Fig. 4c). In the collected urine at T4, the syndecan-1 concentration was significantly reduced compared to T0 (T4: 2.33 ± 0.21 vs. T0: 3.39 ± 0.24 ng/ml, *p* < 0.05, Fig. 4c). After alcohol consumption, the syndecan-1 values in the urine increased again to levels comparable with T0 controls (Fig. 4c). The concentrations of syndecan-1 were significantly reduced in women compared with men at all evaluated time points (*p* < 0.05, Fig. 4d).

### Soluble CD14 in serum

**Fig. 5:**
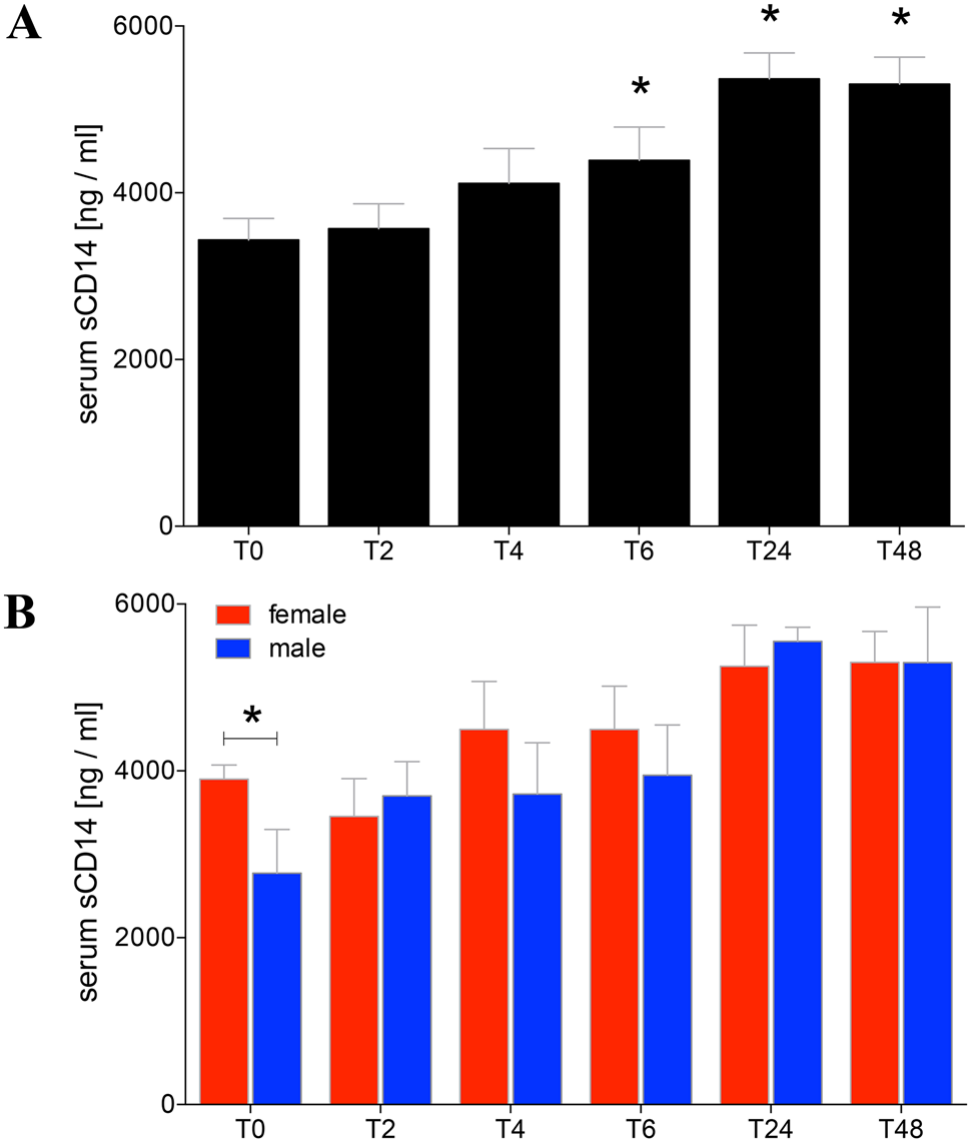
Soluble (s)CD14 in sera before, during and after alcohol consumption. Mean sCD14 values determined were determined in ng/ml in sera from healthy volunteers before (T0), 2 h (T2), 4 h (T4), 6 h (T6), 24 h (T24) and 48 h (T48) after start of alcohol consumption. **a** Data from all healthy volunteers (*n* = 22), and **b** gender-specific results are shown (female: *n* = 12 and male: *n* = 10). The data are presented as mean ± standard error of the mean. *p < 0.05 vs. T0 or in **b** vs. indicated groups

Before alcohol administration, the serum level of sCD14 in healthy volunteers was 3432.00 ± 258.40 ng/ml (Fig. 5a). At T2 and T4, the sCD14 in serum increased continuously compared to T0. At T6, the sCD14 was significantly increased compared to T0 (T6: 4388.00 ± 398.50 vs. T0: 3432.00 ± 258.40 ng/ml, *p* < 0.05, Fig. 5a). The levels of sCD14 remained elevated at T24 and T48 compared to T0 (T24: 5363.00 ± 312.50 or T48: 5300.00 ± 324.20 vs. T0: 3432.00 ± 258.40 ng/ml, *p* < 0.05, Fig. 5a). The gender-specific analyses have shown significant higher levels of sCD14 in women compared to men at T0 (Fig. 5b). In the further time course of alcohol intake there were no significant gender-specific differences (Fig. 5b).

### Correlation of FABP-I and sCD14 in serum

**Fig. 6:**
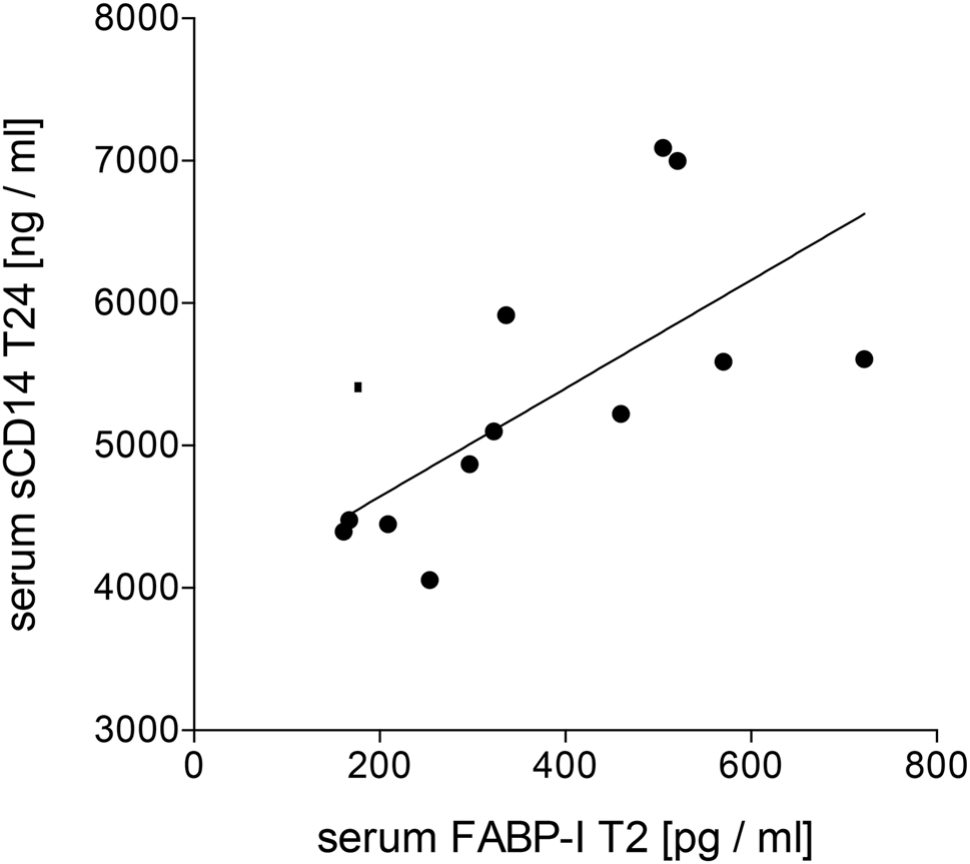
Spearman analysis of the correlation between the systemic soluble (s)CD14 and intestinal fatty-acid binding protein (FABP-I) after acute alcohol consumption. Positive correlation with a Spearman *r* = 0.8182 between the circulatory FABP-I at 2 h (T2) and sCD14 at 24 h after beginning of the alcohol intake is shown (*p* < 0.05, *n* = 12)

The Spearman’s rank correlation showed a significant correlation for systemic FABP-I levels at T2 and sCD14 at T24 (*p* < 0.05, Spearman *r* = 0.8182, Fig. 6). No further correlations were found for other parameters or time points.

## Discussion

The immunological response to acute alcohol intoxication co-determines the clinical course of patients after severe injuries and/or surgical interventions. The existing studies show different results in the outcome of alcoholised patients, but they do not provide exact indications for the underlying cellular mechanism of alcohol-induced immunomodulation. Furthermore, the extent of potentially alcohol-induced damage of the intestinal barrier and its difference in healthy men and women after drinking is not yet known. Therefore, the aim of this study was to evaluate the immunological changes following acute alcohol intake in healthy subjects especially affecting the intestinal barrier in a time- and dose-dependent manner. The concentrations of specific markers of the intestinal barrier integrity FABP-I and syndecan-1 were measured in both serum and urine. Systemic endotoxin activity was evaluated by sCD14 levels.

In our cohort of healthy volunteers was an almost balanced gender distribution. By adjusting the alcohol concentration using the modified Widmark equation, there were no differences in the blood alcohol concentration between both sexes (Fig. 2b). To imitate a typical drinking behavior with corresponding physiological parameters, alcohol intake was induced in all subjects at the same time in the early evening. In addition, the alcohol intake was persisting over a period of hours to simulate the physiological conditions and drinking behavior. The study participants reached already at 2 h after drinking a legally permitted alcohol level (Fig. 2a).

Numerous studies have shown that FABP-I can be used as a non-invasive marker for intestinal diseases like celiac disease [31] or intestinal ischemia [32] and also for intestinal damage in severely injured patients [17]. In the present study, the FABP-I concentration decreased significantly very early after alcohol consumption. However, serum FABP-I concentrations were significantly elevated after 6 h and remains enhanced over the next 2 days (Fig. 3a). The primary decrease of the FABP concentration in serum may be caused by an initially delayed transport and resorption of the alcohol with a full stomach and reduced motility. This decrease in the FABP concentration was mainly present in women, which in turn significantly influence the overall result. The absence of a significant differences among later time points T24 and T48 may be caused by the lower number of included healthy volunteers since several participants did not show up for further blood withdrawals. A similar decrease in urine FABP-I concentration was detected at T4 (Fig. 3c). Serum values indicate damage to the intestinal barrier in the early phase after alcohol intoxication.

Interestingly, it has been shown that severely injured women have a better outcome than men with comparable injury patterns [33]. The underlying mechanism is still unexplained. Hundscheid et al. demonstrated that the small intestine of female is more resistant to reperfusion-related intestinal damage than that of men [34]. Our data show lower serum FABP-I concentration 2 h after alcohol consumption in women, potentially underlining the above-mentioned study results (Fig. 3b). Furthermore, women showed lower syndecan-1 concentrations in both blood and urine before, during and 2 h after alcohol consumption as well (Fig. 4b, d). In addition, higher levels of sCD14 in women under normal conditions compared to men were found (Fig. 5b). In absolute terms, women consumed less volume of alcohol compared to men. However, calculating the amount of alcohol, both height and weight were included resulting in a comparable BAC without differences between both gender.

In non-cirrhotic alcoholics, the intestinal barrier breakdown and systemic LPS were observed and 2 weeks after admission to a detoxification program, the patients still exhibited an increase of proinflammatory cytokines and persistent low-grade-inflammation of intestine [35]. This suggests that alcohol in both acute and chronic settings damages the intestinal barrier and results in increased LPS bioactivity over the period of acute consumption. Afshar et al. have shown an increase in the LPS-induced proinflammatory TNF-α levels 20 min after a single alcohol shot, while after 2 and 5 h an enhanced anti-inflammatory effect due to reduced IL-1β response upon LPS-stimulation appeared [36]. Similarly, Bala et al. administered single vodka mixed drink to healthy volunteers showing a higher BAC in women compared to men. Few hours after alcohol intake, the serum concentration of endotoxin did not change, whereas sCD14 increased significantly after 24 h being in line with our findings [37]. Interestingly, men who fastened 1 day and subsequently consumed a single drink exhibited a significant decrease of medium BAC value (0.07%). On the other hand, de Jong et al. showed, the intestinal FABP increased in serum right from the start of the experiment but there was no change in sCD14 levels [38]. The different results may be based on the differences in the study design. Interestingly, we found a significant decrease in intestinal FABP concentration in serum early after acute alcohol consumption. However, the immediate high-fat nutritional intake before alcohol consumption could have contributed to this result since Lubbers et al. demonstrated that a continuous high-protein and high-fat diet exerts anti-inflammatory effects [39]. We demonstrated that alcohol intake increased serum sCD14 significantly at T6 and remained significantly elevated over the next 2 days at T24 and T48.

SolubleCD14 mediates a complex function with binding of LPS and transfer to the TLR4-receptor complex with subsequent proinflammatory immune response [25], but also due to the possibility of neutralizing LPS [27]. Therefore, sCD14 may serve as a biomarker for LPS bioactivity. The significant increase of FABP-I in serum following acute alcohol consumption suggests a possible damage to the intestinal barrier. This might lead to a potential translocation of pathogen-associated molecular pattern and endotoxins. This may be caused by increased BAC with persisting alcohol consumption and thus also a prolonged direct impact of alcohol itself on the intestine wall. In comparison, Lambert et al. detected systemic endotoxins and TNF-alpha in a mouse study after the animals were administered LPS by intragastric gavage after administration of alcohol [40]. However, it should be noted that the immediate origin of measured sCD14 is not clear, so in addition to shedding from leucocytes, synthesis by hepatocytes is possible, and here sCD14 also occurs as an acute phase protein [41]. Under common circumstances multiple drinks during the evening are usual, and, therefore, our study setting constitutes a realistic scenario providing evidence for a disturbed intestinal barrier function upon drinking. The time line of the barrier loss upon drinking and immune-suppressive effects of alcohol on the other hand must be evaluated in further studies.

### Strengths and limitations

The investigation of the acute alcohol effects on the intestinal barrier and systemic immune response was conducted in a prospective study of healthy volunteers previously examined clinically and by laboratory chemistry. There was an almost balanced gender distribution with no differences in the blood alcohol concentration between the sexes. However, when considering the individual sexes, the divided cohorts into male and female were not particularly large and not all healthy volunteers appeared for the follow-up measurements at t24 and t48. Furthermore, concomitant effects due to the previous food intake and the use of a mixed drink with cola cannot be excluded. Although our study design is intended to illustrate a realistic scenario with food and several drinks taken over the evening.

## Conclusion

We investigated the time-dependent effect of acute alcohol consumption of one per mille on the intestinal barrier in 22 healthy volunteers. 6 h after the onset of alcohol consumption, a significant increase of FABP-I was detectable in serum, suggesting damage to the intestinal barrier after acute alcohol consumption. Furthermore, sCD14 was shown to be significantly elevated at T6, T24 and T48. This suggests an increased LPS bioactivity due to the influence of acute alcohol drinking potentially causing damage of the intestinal barrier, which is still present 2 days after alcohol ingestion. Interestingly, gender-specific effects were observed although the same BAC was present. Females exhibit significantly lower syndecan-1 levels and significantly higher serum sCD14 levels in blood before alcohol intake.

After acute alcohol consumption a potential damage to the intestinal barrier and presumed enhanced systemic endotoxin bioactivity is suggested, which may represent a continuous immunological challenge to the organism and which should be considered for the following days after drinking.
